# Ankle Bracing as a Public Health Game Changer: A Narrative Review on the Prevention of Ankle Injuries in Basketball Players

**DOI:** 10.3390/medicina62020287

**Published:** 2026-01-31

**Authors:** Goran Slivšek, Marin Marinović, Sandra Mijač, Ivan Dolanc, Silvija Petković, Renato Mautner, Josip Kranjčić, Iva Sorta-Bilajac Turina, Karmen Lončarek, Ksenija Vitale, Miran Čoklo

**Affiliations:** 1 Institute for Anthropological Research, 10000 Zagreb, Croatia; miran.coklo@inantro.hr; 2 Faculty of Medicine, University of Zagreb, 10000 Zagreb, Croatia; sandra.mijac@vip.hr (S.M.); renmautner@gmail.com (R.M.); ksenija.vitale@snz.hr (K.V.); 3 Faculty of Medicine, University of Rijeka, 51000 Rijeka, Croatia; marin.marinovic2@gmail.com (M.M.); silvija.petkovic@skole.hr (S.P.); iva.sorta-bilajac@zzjzpgz.hr (I.S.-B.T.); karmen.loncarek@uniri.hr (K.L.); 4 Rijeka University Hospital Centre, Krešimirova 42, 51000 Rijeka, Croatia; 5 Children’s Hospital Srebrnjak, 10000 Zagreb, Croatia; 6 School of Dental Medicine, University of Zagreb, 10000 Zagreb, Croatia; kranjcic@sfzg.unizg.hr; 7 University Hospital Dubrava, 10000 Zagreb, Croatia

**Keywords:** ankle, ankle injuries, athlete, basketball, braces, physical activity, prevention, public health, social inclusion, sports injuries

## Abstract

Ankle injuries are among the most common sports injuries in basketball and represent a substantial public health and economic burden. This narrative review synthesises evidence on ankle bracing as external protective support and shows that ankle braces reduce the risk of both first-time injuries and ankle re-injuries in basketball players without significantly affecting sport-specific performance, such as sprinting, jumping, or changing direction. Similarly, despite earlier theoretical concerns, current evidence shows no increased risk of knee injury associated with the use of ankle bracing. Mechanistic studies indicate that protection is provided by limiting excessive frontal-plane motion, enhancing proprioceptive feedback, and increasing perceived joint stability. Economic analyses show that a single ankle injury generates considerable direct and indirect costs, whereas seasonal team-wide ankle bracing programmes are low cost per athlete and likely cost-effective at scale. As a public health measure, ankle bracing is practical and easily scalable in community and sports settings. Overall, routine ankle bracing is shown to be a feasible, low-cost strategy for primary and secondary prevention of ankle injuries in basketball without compromising performance, and may support broader participation goals aligned with Sport for All principles.

## 1. Introduction

Engagement in sport is widely recognised as a powerful driver of personal growth, cultivating determination, self-discipline, healthy habits, and effective teamwork. Beyond performance, regular physical activity enhances overall well-being by improving health, reducing disease risk, providing purposeful leisure, and fostering relaxation and social connection among people. Although it is clear that playing sports offers numerous benefits, it also carries the risk of sports injury [1]. Basketball is generally considered one of the non-contact sports with the highest risk of sports injury, particularly ankle injuries [2,3,4,5]. Several studies have shown that basketball has one of the highest rates of sports-related injuries compared to many other sports, with some suggesting this risk may equal or even exceed that of certain full-contact sports [6,7,8,9,10,11,12,13]. This is unsurprising, given the nature of basketball, which involves rapid acceleration, frequent changes of direction, and repeated jumping and landing, all of which increase the likelihood of awkward landings, collisions, and falls [14].

Sports injuries in basketball can be broadly categorised as acute or chronic. Acute sports injuries usually occur suddenly during play, often due to falls, collisions, or awkward landings, resulting in sprains and other traumatic outcomes [15]. In contrast, chronic sports injuries develop from prolonged overuse or strain on specific body parts caused by repetitive training and competition [14]. Common acute sports injuries in basketball include fractures, sprains, and dislocations, while chronic conditions such as tendinopathy, plantar fasciitis, and bursitis are often linked to repeated loading and insufficient recovery [16,17]. A common mechanism of acute sports injury is exposure to external loads that exceed tissue tolerance, for example, during player contact, sudden deceleration, or awkward landings. Acute sports injuries can result not only from direct contact but also from overexertion, which may contribute to chronic conditions over time [18,19]. The repetitive nature of basketball movements, including jumping, landing, and rapid changes of direction, can lead to chronic sports injuries that may cause long-term health problems [4,20]. The most common sports injuries in basketball are injuries to the lower limbs, especially ankle, knee and lower back injuries [21,22,23]. The ankle is prone to sprains due to very rapid changes of direction and jumping, and the knee is often injured by turning, stopping and jumping, while the lower back can be more susceptible to injury due to sudden movements, jumping and hitting during the game [4,24]. Many studies have shown that ankle injuries are most common in basketball players, regardless of whether they play at school or professional level, and the most common injury is a sprained ankle [13,25,26]. A study conducted by Leanderson et al. showed that 92% of basketball players had suffered an ankle sprain during the game, and 83% of them reported a previous ankle sprain [27]. Accordingly, other similar studies have shown that up to 60% of people who play basketball sprain their ankle repeatedly [28]. Once an ankle is sprained, up to 80% will sprain it again, and people who play basketball are about five times more likely to re-injure their ankle after a previous injury [29]. Re-injuring the ankle can lead to long-term problems such as instability and reduced mobility, increasing the risk of further injury and various health complications. Ultimately, this could lead to permanent disability and affect overall quality of life [30,31].

From a public health perspective, sports injuries require a prevention-focused approach, as even modest relative reductions can lead to substantial absolute health and economic benefits at the population level, particularly in widely practised sports such as basketball [32,33,34,35,36,37]. In modern sports medicine, prevention has received increasing attention, supported by significant advances in understanding sports injury development and identifying modifiable intrinsic and extrinsic risk factors [38,39,40,41]. Preventive measures can reduce sports injury risk by more than 50%, but real-world effectiveness depends on implementation, including systematic injury recording and monitoring, which may be improved by enhanced surveillance and sensor-based technologies [38,42,43,44,45,46]. The economic rationale is reinforced by the broader burden of sports injuries, as European data indicate substantial healthcare expenditure attributable to sports injuries, and national estimates suggest that annual costs in the Netherlands alone exceed three billion euros [34,36]. Quantifying the direct and indirect costs of ankle sprains, along with the associated time lost from sport, therefore provides an important basis for evaluating the cost-effectiveness of targeted preventive measures [47,48]. However, it is estimated that ankle sprains have an average financial cost, with direct costs ranging from 262 to 2032 euros per person and indirect costs ranging from 1328 to 3890 euros, exacerbated by a typical absence from the playground of approximately 21 to 30 days. In addition, the increased risk of secondary injuries and reduced training time further increase the overall cost of such a sports injury [25]. Compared to that, the costs associated with the implementation of preventive measures are relatively low, typically averaging less than 100 euros per player per season [49,50]. Based on the latest data, around 11% of the world’s population, or over 450 million people, currently play basketball either professionally or at amateur level [51,52]. Given that basketball is the second most popular sport in the world after football [53,54], it is clear that cost savings from the prevention of sports injuries in basketball would have a significant impact on the overall expenditure associated with such injuries [55].

It has been shown that there are three basic effective methods to prevent sports injuries to the ankle in basketball players: proprioceptive training, strength training and the use of protective external supports such as braces, taping or orthoses [56]. Proprioceptive training improves the body’s awareness of joint position and movement. This type of training typically includes activities such as the one-legged stance, the use of a balance board or cushion, and exercises that focus on improving the stability of the ankle joint. These activities help to strengthen the ankle muscles, increase flexibility and ultimately reduce the risk of injury [57,58]. Strength training can strengthen the ankle muscles, including the calf, calf and foot muscles, which can contribute to greater ankle stability. This usually involves squats, lunges, exercises on machines or the use of free weights [59]. The use of protective external supports such as braces, taping or orthoses can provide additional support to the ankle during basketball play. This can contribute to greater stability of the ankle and reduce the risk of extreme movements that can lead to injuries such as sprains [60]. While braces, taping, and orthoses all serve as protective external supports intended to enhance stability and prevent sports injuries, it is important to distinguish between these interventions. Taping is generally considered a separate method in sports injury prevention. Specifically, ankle taping involves applying adhesive tape, either rigid or elastic, to provide temporary protective external support. In contrast, ankle braces (soft, semi-rigid, or rigid) and orthoses are wearable devices designed to offer more consistent and reusable protective external support for the ankle [61,62,63,64,65].

**Figure 1:**
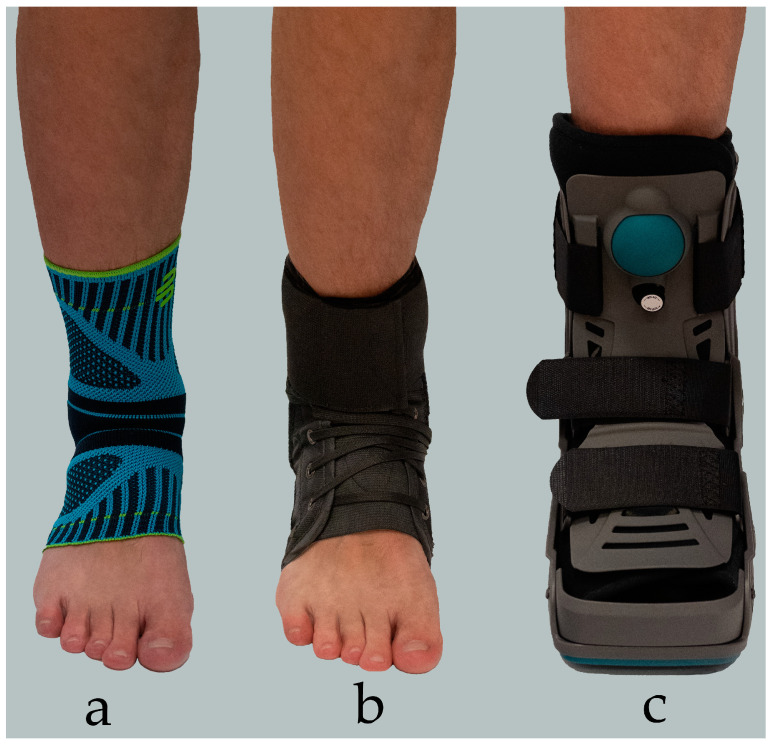
Three Main Types of Ankle Braces: (**a**) Soft Ankle Brace; (**b**) Semi-rigid Ankle Brace; (**c**) Rigid Ankle Brace.

Ankle braces come in various forms, and each of them helps to some extent to create a balance between stability and mobility of the ankle, with greater mobility allowing for less stability and vice versa [65]. In terms of the degree of support, three main groups of ankle braces can be distinguished: soft braces/supports, semi-rigid braces, which can be lace-up or hinged, and also rigid braces (Figure 1) [64].

Each of them is designed to reduce the range of movement of the ankle in the frontal plane (inversion/eversion) and sometimes also in the sagittal plane (dorsal/plantar flexion) [66]. Their selection depends mainly on the athlete’s needs, the type of injury, the level of support required and the planned activities (Table 1) [67].

It is now known that all these different methods effectively reduce the number of ankle injuries in athletes, especially basketball players. Today, taping, which may be soft (elastic) or rigid (non-elastic), is also widely used as a protective external support alongside braces and often as a potential alternative to bracing [63]. Soft taping is generally more comfortable and less restrictive, providing support that varies during activity. However, its stabilising effect often decreases during activity, particularly with higher intensity or prolonged effort, as the material stretches and loses tension after the initial application. The effects are primarily sensorimotor, characterised by a limited and variable mechanical constraint that depends greatly on technique and is generally less pronounced than those of rigid taping [65]. In contrast, rigid taping provides immediate mechanical restriction and stabilisation of the ankle joint, primarily in inversion and eversion, and to a lesser extent in plantarflexion and dorsiflexion, although this restriction also decreases with exercise [62,68]. In clinical settings, soft taping typically uses kinesio taping (KT), elastic adhesive bandage (EAB), and dynamic tape (DT). In the case of rigid taping, it is most often performed with zinc oxide strapping tapes such as Leukotape P, Strapped, and Mueller MTape, while alternatives with acrylic, synthetic, or other adhesives that offer comparable strength and adhesion are used when skin tolerance or clinical factors require it [60,69,70]. The choice between soft and rigid taping depends primarily on the athlete’s needs, the specific injury, the required level of support, and the demands of upcoming activities (Table 2) [63,65].

Protective external supports such as braces, taping or orthoses are most commonly used for primary or secondary prevention of ankle injuries in basketball players [61,71,72]. Primary measures to prevent ankle injuries in basketball players aim to prevent injuries before they occur, while secondary measures aim to prevent re-injury and reduce the effects of an ankle injury that has already occurred [72]. Dizon et al. found that the use of ankle braces and taping can reduce the risk of ankle sprains, which are among the most common sports injuries and occur nearly seven times more frequently than other ankle injuries, by approximately 69% with bracing and 71% with taping in previously injured athletes, but neither method was superior [61,73,74]. Based on a comprehensive review and analysis of the latest relevant literature, this narrative review aims to explore the effectiveness of ankle braces in the prevention of ankle injuries in basketball players compared to not using this preventive measure.

## 2. Materials and Methods

A comprehensive search was conducted between December 2024 and February 2025 across major international medical bibliographic databases, including Scopus, Web of Science Core Collection (WoS CC), Embase, and MEDLINE, with the final search on 28 February 2025. Keyword combinations included “basketball”, “ankle injury”, “ankle sprain”, “ankle brace”, “external support”, “taping”, “stretching” and “prevention”, combined using the Boolean operators AND/OR. Searches were limited to English-language records with at least an English abstract or summary, covering the period from 1973 to 2025. The year 1973 was chosen because, according to the PubMed search engine, it is the earliest available study exploring the preventive role of ankle bracing as external support against ankle injuries in basketball players. Records were de-duplicated in EndNote 2025 (Clarivate Analytics, London, UK) and managed for screening in Microsoft Excel 365 (Microsoft Corporation, Redmond, WA, USA).

Eligible studies included human athletes from youth to professional levels that examined external ankle supports in the form of braces, with or without comparison to taping or no support, and reported at least one relevant outcome, such as ankle injury, ankle re-injury, performance measures, biomechanical or neuromuscular parameters, or adverse events related to the ankle. Included designs comprised randomised and non-randomised interventions, prospective cohort studies, and laboratory or biomechanics investigations, while systematic reviews and meta-analyses were used for contextualisation. In mixed-sport cohorts, basketball-specific outcomes were extracted when distinguishable, while results that could not be separated were referenced solely for mechanistic context. Editorials, expert opinions, and single-case reports without generalisable data, as well as studies in non-sport or postoperative populations, were excluded unless they provided mechanistic insights directly relevant to ankle injury prevention with protective external supports using bracing, while grey literature was completely omitted. Where overlapping cohorts were identified, the most comprehensive or most recent report was prioritised. To ensure as much comprehensive coverage as possible, the snowballing technique was applied by systematically reviewing the reference lists of all included studies and performing backward and forward citation tracking to identify additional relevant records.

Two reviewers independently screened titles, abstracts, and full texts against predefined eligibility criteria, resolving disagreements by consensus with a third reviewer, while a fourth researcher verified the accuracy and completeness of data extraction. As this was a narrative review, no formal quantitative risk-of-bias tool was applied. Instead, studies were qualitatively appraised, with attention to exposure metrics such as athlete exposures or game and practice time, study design hierarchy, clarity and consistency of outcome definitions, and compliance with bracing protocols. Although publication bias was not quantitatively assessed due to the narrative review design and the heterogeneity of the included study designs, selection bias was mitigated through predefined eligibility criteria, independent screening, multiple database searches, and systematic snowball citation tracking. Furthermore, two independent reviewers assessed the risk of bias for each eligible study across predefined domains. Each domain was rated as having low risk of bias, some concerns, or unclear risk. Disagreements were resolved through discussion or, if necessary, by consulting a third reviewer. To ensure adherence to this methodology, studies assessed as having a high risk of bias were either excluded from this narrative review or, if retained, were interpreted with particular caution and did not affect the main conclusions. To further reduce publication bias and increase transparency, the protocol for this narrative review was preregistered on the Open Science Framework (OSF: xzvu4). The collected articles were comprehensively reviewed, and a narrative literature review was compiled [75,76].

## 3. Thematic Synthesis: Topics and Results

Through this thematic synthesis, the reviewed narrative evidence is examined within the framework of sports medicine, which closely aligns with the core principles of public health. In this context, sport is regarded not only as a field of performance but also as a platform for inclusion, prevention, and the support of lifelong physical and psychological well-being [32,77,78]. The following sections integrate insights from epidemiology, biomechanics, and health policy to examine how accessible participation and structured preventive strategies can promote safer environments, reduce the risk of ankle injury among basketball players, and incorporate the hallmarks of Sport for All (SFA).

### 3.1. Sport for All: Inclusive Sports and Preventive Measures

Physical activity and sports offer a wide range of benefits for human health, encompassing both mental and physical well-being [79,80]. This fact, among others, has contributed to the development of SFA, a global movement inspired by the Olympic idea that promotes access to physical activity and sports for people of all ages, abilities and backgrounds as a human right that must be accessible to all [81,82]. It emphasises the importance of breaking down barriers to participation in sports and ensuring that everyone has the opportunity to participate in physical activities that contribute to overall health, well-being and social inclusion through sports. By focusing on promoting broad participation in sport, SFA seeks to create more inclusive, active and healthy communities worldwide [83]. Therefore, the World Health Organisation (WHO), as the umbrella organisation responsible for international public health, ensures access to movement SFA through its health programmes. SFA in its settings has inclusion, which implies fostering an environment where different perspectives, backgrounds, experiences and identities are valued and everyone feels included, valued and empowered [83,84]. It goes beyond mere tolerance or diversity and emphasises the active participation and engagement of all individuals, creating a sense of belonging, respect and support for everyone in a group or community, regardless of their differences. Through inclusive policies and community-led initiatives, SFA aims to make physical activity a universal right [85]. Inclusive sports promote social inclusion by providing equal opportunities for people with different abilities, backgrounds, and identities to participate. By breaking down barriers and promoting unity, diverse communities come together through shared sporting experiences, leading to a more cohesive and inclusive society [83,85,86]. Social inclusion in sports is based on the social model that advocates SFA, which ultimately leads to greater self-confidence, creating empowerment and mixed ability sports that makes sports and physical activity accessible to everyone [87,88,89,90,91,92].

The fear of injuring or re-injuring prevents some people, especially those with disabilities, from engaging in physical activity and sports [93,94,95,96]. Fischerauer et al. revealed that fear avoidance is independently associated with reduced physical function in injured athletes. Even after adjusting for other variables, such as pain catastrophising, fear avoidance continued to have a notable and separate effect on reduced physical function and thus on sports participation [97]. On the other hand, people with disabilities, whether in amateur or professional sports, are often exposed to a higher risk of sports injuries due to their physical limitations, which can lead them to reduce their physical activity and thus also their sports [98,99]. This is in line with the study conducted by Sretenović et al., which showed that 76.6% of para-athletes had suffered at least one injury, 63.3% of which occurred during training, the majority of which was trauma [100]. Therefore, people who play sports and thus engage in significant physical activity, whether as amateurs or professionals, can greatly benefit from preventive measures that effectively reduce the risk of sports injuries during physical activity through sports, especially if they have a disability [101,102,103]. Basketball is consistently associated with a substantial sports injury burden, with ankle injuries comprising a significant proportion, making it a relevant setting for SFA [13,50,51,55]. Preventing ankle injuries in basketball can be considered an enabling factor for sports inclusion, as reducing common time-loss injuries supports confidence, continued participation, and long-term engagement in sport, all of which align with SFA aims. People with disabilities may be more susceptible to sports injuries, and these injuries may further hinder participation, highlighting the need for accessible and effective public health preventive measures [77,98,99,100,101]. Ankle bracing is an evidence-based preventive measure and aligns with SFA in practice due to its feasibility and scalability across schools, clubs, and community programmes, including settings with limited medical resources, and can be implemented as a routine preventive strategy [62,67,89]. Ensuring access to ankle bracing and providing targeted education for players, coaches, and support staff on brace selection, fitting, and adherence can enhance safety and promote sports inclusion in high-injury sports such as basketball, supporting broader public health goals of reducing injury burden and advancing health equity [33,48,77,104,105]. Promoting preventive measures in sports improves safety and makes sports more accessible to all, including people with disabilities. These measures align with the values of the SFA movement and ensure that everyone, regardless of ability, can safely participate in and enjoy sports [106]. This underscores the critical need for inclusive practices that support the prevention of sports injuries, such as the use of protective external supports such as ankle braces to prevent ankle injuries in basketball, as preventive measures [56,72]. By ensuring that these protective measures are accessible to all people who participate in sports, regardless of ability, public health initiatives can create a safer and more inclusive environment for participation in sports, particularly in sports with a high injury rate, such as basketball [13,107,108]. This approach can not only enable people with disabilities to participate confidently in physical activities, but also emphasises the broader public health goal of reducing the number of sports injuries, which are a significant public health problem today [109]. Preventive measures appear to be an important tool for promoting health equity by ensuring that everyone can safely participate in physical activities through sports [17,104].

**Figure 2:**
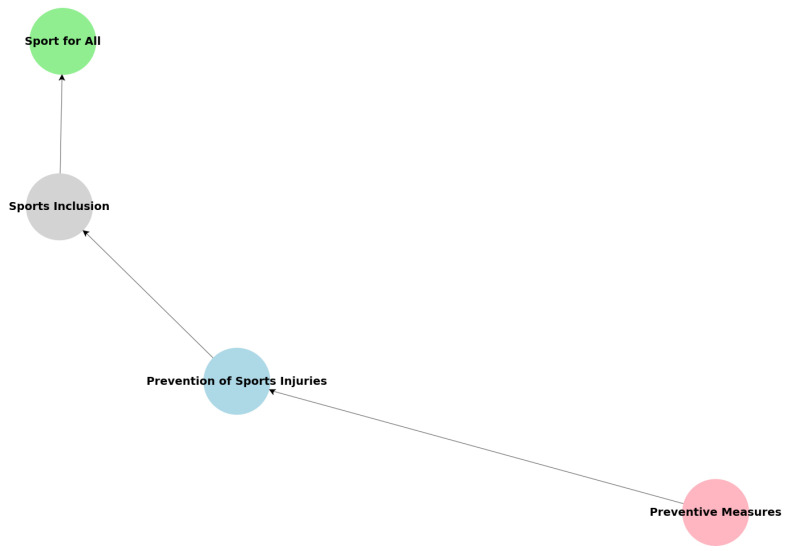
Pathway to Inclusive Sports: From Prevention to Participation.

It is, therefore, clear that preventive measures lead to the avoidance of sports injuries, which in turn promotes sporting inclusion by making sports safer and more accessible for all, including people with disabilities. Ultimately, sports inclusion is a crucial part of the SFA movement, which aims to ensure that all people, regardless of ability, can safely participate in physical activity, promoting overall health and well-being (Figure 2) [77,105,110].

### 3.2. Ankle Bracing and Prevention of Ankle Injuries in Basketball Players

**Figure 3:**
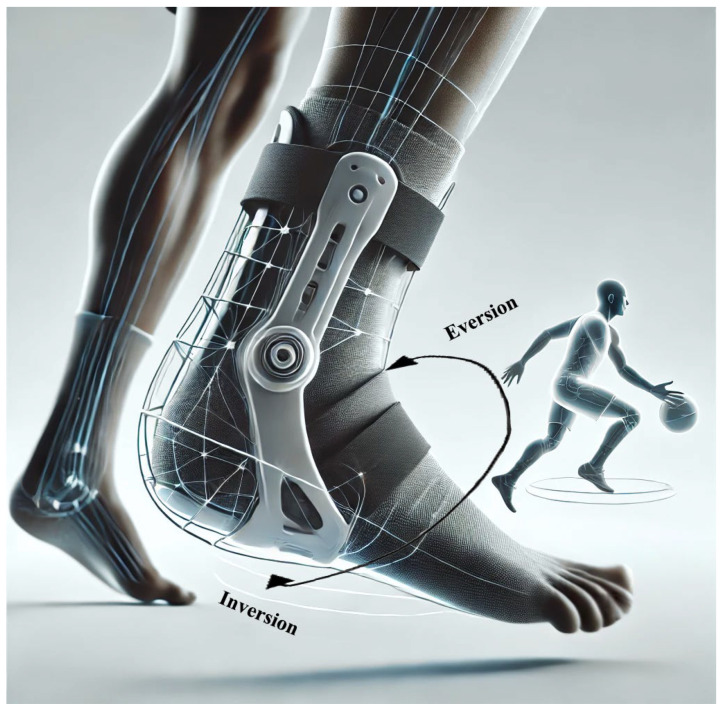
Possible Protective Mechanisms of Ankle Braces: Restriction of the Range of Motion of the Ankle, Limitation of Excessive Inversion or Eversion Movements and Stabilisation of the Ankle.

It is common to see basketball players wearing ankle braces on their own, suggesting that their benefits are widely recognised. Ankle braces provide external support to the ankle joint and improve its stability during dynamic movements such as jumping, cutting and twisting, which are common in basketball [111]. The ability of ankle braces to limit excessive inversion or eversion movements, which often lead to sprains due to stretching or tearing of the ligaments, is the main reason for their effectiveness in reducing ankle injuries (Figure 3) [64,112].

From a pathophysiological perspective, ankle injuries frequently occur during rapid cutting, landing, or directional changes that impose significant torsional and compressive forces on the ankle [63,65,113]. These forces can cause excessive inversion of the ankle, frequently at velocities that exceed the time available for protective neuromuscular responses, resulting in ankle sprains [60,114]. This leads to ligament strain that may surpass tissue tolerance, causing micro-failure and, in more severe cases, macroscopic tearing. Ankle bracing may reduce both initial injury and re-injury risk through several complementary mechanisms [113,115]. It is assumed that ankle braces restrict the ankle’s range of motion (ROM) in these vulnerable directions and thus prevent the ankle from moving beyond its natural range [60,62]. Mechanical stabilisation is the most direct mechanism, as ankle braces provide external constraint that limits end-range frontal plane motion and helps maintain functional alignment during high-risk tasks [116]. Depending on ankle brace design, some restriction of sagittal plane motion may also occur [65]. By limiting abnormal joint displacement, ankle bracing can reduce peak local strain on the lateral ligament complex and distribute external loads more evenly across periarticular structures, which may lower the probability of overstretching during perturbations. Some ankle brace designs may also assist pre-contact positioning of the foot and ankle and provide direct mechanical resistance to sudden inversion moments [64,117,118]. These mechanisms are not mutually exclusive, and their relative contribution likely depends on ankle brace design, task demands, and athlete-specific factors. Neuromuscular and proprioceptive effects may further contribute, as cutaneous stimulation and external support can enhance joint position and movement sense, supporting stabilising responses to unexpected perturbations, particularly during unfavourable landings or twisting events [114,119,120]. Dynamic muscular support and fatigue-related control loss may be relevant during prolonged play, as sharing stabilising demands with the peroneal musculature may reduce fatigue-related decrements in motor control that predispose athletes to recurrent sprain events [65,119,121]. By providing external support, an ankle brace relieves some of the load that these muscles normally bear in maintaining stability, reducing the likelihood of muscle fatigue and subsequent loss of control of the ankle joint. This muscle relief not only prevents immediate sports injuries such as sprains, but also reduces the risk of chronic overuse conditions such as tendinitis, which can result from repetitive strain on the muscles and tendons around the ankle and lead to chronic sports injuries. Finally, ankle braces help to align the foot and ankle joint in the frontal plane before initial ground contact or provide mechanical resistance against inversion [119,120].

Several studies have shown that using ankle braces or tape can significantly reduce the risk of ankle injuries among basketball players. Garrick et al. demonstrated that rigid ankle taping as protective external support reduced the overall incidence of ankle injuries, particularly ankle sprains, by over 55% compared to no taping, and lowered the risk of re-injury by approximately two-thirds [122]. Accordingly, Sitler et al. observed that the use of a semi-rigid ankle brace can significantly reduce the incidence of ankle injuries in basketball players and that the attitude towards the use of ankle braces to prevent ankle injuries improves significantly with increasing use [71]. Karlsson et al. concluded that using ankle braces in sports such as football, handball and basketball can significantly reduce the frequency of ankle injuries such as sprains, especially in people with a previous history of ankle injuries [123]. Similarly, Moiler et al. found that rigid ankle taping had a protective effect against ankle injuries in basketball players [124]. This is not surprising, as ankle taping is commonly used as an alternative to ankle bracing for protective external support, and some athletes perceive taping as less restrictive than certain types of ankle braces, while still providing temporary protective external support for the ankle [63,69]. The study by McGuine et al. emphasises the effectiveness of ankle braces in reducing ankle injuries in basketball players, regardless of whether they were previously injured. Specifically, they observed that wearing an ankle brace reduced the risk of ankle injury by up to 70% in basketball players with no previous history of ankle injuries [125].

According to Farwell et al., there is moderate evidence that ankle braces are beneficial for athletes, especially football and basketball players. Level B evidence suggests that ankle braces are effective in reducing the risk of ankle injuries in athletes [126]. Additionally, Crockett et al. found out that wearing an ankle brace in basketball can lead to a significant improvement in dynamic postural control and functional performance in athletes [127]. A study carried out by Klem et al. also showed that the use of ankle braces can significantly reduce the risk of ankle injuries when playing basketball [64]. Castro et al. found that wearing an ankle brace can improve the protection of the athlete’s body by reducing shear forces during a basketball jump. Their results suggest that wearing an ankle brace has no significant effect on propulsive forces during takeoff or the quality of shock absorption during landing, indicating its potential effectiveness in preventing ankle injuries in basketball players [128]. More recently, Castro et al. demonstrated that wearing an ankle brace reduced mediolateral ground reaction force (GRF) peaks by approximately 9–16% compared with no brace, while vertical ground reaction force (vGRF) variables and jump height remained unchanged. This further supports the protective effect of ankle bracing without significantly compromising performance [129]. A study by Bellows et al. fleshes out that an ankle brace reduces the frequency and relative risk of ankle sprains in athletes such as basketball, football, and volleyball players [130]. Dewar et al. pointed out that wearing ankle braces in basketball players significantly reduces ankle inversion. Specifically, the maximum inversion of the foot is reduced by about two to three degrees, which helps to prevent sudden movements in the end range that can cause ligament damage during inversion. In addition, the use of ankle braces also reduces the strain on the ligaments and muscles of the ankle in the event of ankle injuries [118]. Consistent with this, Aarts et al. highlighted that ankle injuries in basketball players can be reduced by wearing a lace-up ankle brace, and Dizon et al. noted that there was no significant difference in the effectiveness of ankle injury prevention with respect to the type of protective external support when considering different groups of ankle braces [61,131]. However, numerous studies show that ankle braces still provide significantly better protective external support, are easier to use and are more effective in preventing ankle injuries than, for instance, KT [132,133,134]. Some studies have also established that bracing could be a more cost-effective option for sports programmes to prevent ankle injuries in basketball, as it saves both time and money compared to KT [63,135]. This is further supported by a study conducted by Olmsted et al., which indicates that both taping and bracing effectively prevent ankle sprains, particularly in athletes with a history of re-injury, and that bracing provides equivalent or superior protection at approximately one-third the cost of taping [73]. These results also align with a study by Romero-Morales et al. which suggests that the initial application of KT to the ankle leads to a reduction in the range of movement of dorsiflexion in basketball players. However, the study implied that the effectiveness decreases significantly after 30 and 90 min, emphasising the potential need for semi-rigid ankle brace to achieve a more sustained preventive effect on ankle injuries [68].

Hall et al. explain the effectiveness of ankle braces in preventing ankle injuries by influencing range of movement and perception of stability [62], which is not unexpected as the ankle brace provides significant protective external support to the ankle [67,136]. Ankle braces not only offer physical protection, but also have a significant psychological effect. In basketball, they can give players a sense of confidence in their movements and overall performance. When athletes feel that their ankles are safe, they are less plagued by the fear of re-injury, leading to improved performance and a reduced likelihood of compensatory movements that could potentially cause other injuries [62,137,138]. The pressure itself, which is a consequence of applying an ankle brace, can also contribute to a reduction in swelling and thus to the development of inflammation and pain [139,140]. Pienkowski et al. proved that wearing an ankle brace, which is intended to provide protective external support and prevent ankle injuries, does not impair athletic performance. This result brought out the importance of wearing ankle braces in sports such as basketball, where ankle injuries are common, and allows athletes to play more safely without compromising their athletic ability [141]. A similar finding was confirmed by Head et al., who showed that ankle bracing does not adversely affect key athletic performance metrics and can serve as an effective primary and secondary preventive measure against ankle injuries [61,72,125,142]. Recent studies show that wearing ankle braces in basketball players does not lead to an increased risk of knee injuries, as previously assumed. It was found that changes in landing forces and knee movements are not affected by the use of ankle braces, which contradicts the previous assumption [71,125,143]. Although there is some concern about the possible delayed activation of muscles associated with wearing an ankle brace, studies have found no effect on the activation of individual muscles with prolonged wear, disproving previous suggestions that ankle braces could cause muscle weakness [114,120,121]. Namely, in recent years, considerable advances have been made in the technology of ankle braces, and this progress has also contributed significantly to changing the earlier opinion that ankle bracing should be avoided due to possible side effects [144]. A study by Castro et al. also fits in with this, which has shown that the use of an ankle brace does not affect the ability of the ankle muscles to generate dynamic torque or the functional balance of the ankle. This ultimately means that the use of an ankle brace does not affect the strength of the ankle muscles during a basketball game, which is in line with similar studies [145].

The use of ankle braces to prevent ankle injuries in basketball players is proving to be a cost-effective public health preventive measure. Investing in ankle braces can significantly reduce the costs associated with injury treatment and recovery, as fewer medical interventions are required and recovery does not take as long. This not only ensures the health and safety of players, but also reduces the overall cost of the healthcare system, contributing to the profitability and sustainability of sports activities [146,147]. The study conducted by Kaminski et al. has revealed that the use of protective external supports is very effective in preventing both first-time injuries and re-injuries to the ankle. The use of ankle braces proved to be perhaps the best solution in terms of reducing costs and risks as a successful approach to reducing ankle injuries in sports, especially those such as basketball [148].

From all this, it is clear that the application of ankle braces is an effective preventive measure for the prevention of ankle injuries in basketball players, whether as a primary or secondary preventive measure. Therefore, it is very important to promote the use of ankle braces in basketball players to prevent ankle injuries, more specifically, to remove the barriers to the use of these ankle braces. Studies have shown that the most common barriers to the use of protective external supports to prevent ankle injuries are feelings of discomfort and the impression that they are not necessary. However, it is not entirely clear what actually influences basketball players’ decision to use any type of protective external support, including an ankle brace [149]. In general, it is known that parental influence, younger age and a sense of security have a significant positive effect on the increased use of protective external supports, while discomfort seems to be the main reason for non-use [150,151,152]. Cusimano et al. discovered that basketball players were more inclined to use external protective supports, such as ankle braces, to prevent ankle injuries if they were encouraged to do so by their coaches and if they considered ankle injuries to be serious. The study also revealed that coach influence and a history of ankle injury had the strongest influence on the use of protective external supports. Interestingly, the aesthetics of protective external supports such as the ankle brace itself have been the biggest obstacle to their use in the prevention of ankle injuries in basketball players [149]. Consistent with McKay et al., slightly more than two-thirds of basketball players who sustained an ankle injury reported using protective external supports upon returning to competition, most commonly some type of taping or brace. Among basketball players with a previous ankle injury, taping was associated with a trend towards lower re-injury risk, suggesting it may be a promising secondary preventive measure. However, this predominantly reactive use of protective external supports for ankle injuries also highlights the need for proactive, preventive implementation, particularly among basketball players with known risk factors for ankle injuries [153]. Numerous comprehensive studies indicate that promoting and optimising the use of ankle braces as a proactive external support can significantly improve the safety and overall health of basketball players. This helps to minimise the risk of ankle injuries, as ankle braces support the ankle so that it remains stable and cannot twist as easily. This allows players to concentrate better on their performance during the game [120]. Table 3 presents a quantitative summary of key evidence on the use of protective external supports in basketball for the prevention of ankle injuries.

## 4. Conclusions

Evidence indicates that ankle bracing reduces the incidence of both first-time ankle injuries and re-injuries in basketball, regardless of ankle brace type, without consistent or clinically meaningful reductions in performance. In addition to mechanical stabilisation, ankle braces also provide psychological reassurance that supports on-court performance, and current evidence does not indicate an increased risk of knee injury associated with ankle bracing. Ankle bracing is cost-effective, is widely accessible, and can be implemented immediately. This narrative review situates ankle bracing within a public health framework and highlights a practical prevention approach that combines routine ankle brace use with a warm-up focused on dynamic stretching, while static stretching is kept brief and preferably reserved for the cool-down or, if used before activity, is followed by activation exercises to preserve performance. Given the high burden and cost of ankle injuries in basketball, broad adoption of ankle bracing can reduce healthcare expenditure and long-term disability, which is often associated with sports injuries, especially in basketball. Therefore, ankle bracing can be a safe, effective, and economical public health measure for both primary and secondary prevention across school, recreational, and professional settings, aligned with SFA goals.

This study clarifies three points that extend previous reviews on this topic: routine ankle bracing does not increase the risk of knee injury, does not cause consistent performance decrements, and serves as a scalable, cost-effective public health measure. It also translates these insights into practice by outlining an implementation pathway for routine ankle brace use and providing clear, actionable steps to promote uptake across schools, clubs, and athletic programmes, supporting sustained prevention of sports-related ankle injuries among basketball players.

## Figures and Tables

**Table 1:** Comparison of Ankle Brace Types and Their Characteristics.

Type	Material	Comfort	Mobility	Support Level
soft	elastic materials, neoprene	high	high	low to moderate
semi-rigid	soft materials with rigid components	moderate	moderate	moderate to high
rigid	hard plastic, carbon fibre, metal	low to moderate	low to moderate	high to maximum

**Table 2:** Comparison of Ankle Taping Types and Their Characteristics.

Type	Material	Comfort	Mobility	Support Level
soft or elastic	elastic adhesive or cohesive	high to very high	high to very high	low to moderate
rigid or non-elastic	non-elastic with some adhesive	low to moderate	low to moderate	moderate to high

**Table 3:** Quantitative Summary of Key Evidence on Protective External Supports in Basketball for the Prevention of Ankle Injuries.

Leading Reference First Author Year	Type of Study	Type of Protective External Supports	N (f + m) Age in Years	Research Area	Outcome	Statistical Data
Garrick 1973 [122]	prospective controlled cohort	ankle taping and shoe height	2562 college-aged	primary and secondary prevention	ankle taping significantly reduced the incidence of ankle sprains and markedly lowered the risk of re-injury, whereas high-top shoes offered additional protection	All rates are reported as ankle sprains per 1000 player-games (taped vs. untaped): overall incidence density 14.70 vs. 32.80 first-time ankle sprains 10.90 vs. 17.90 re-injury 22.10 vs. 140.00 high-top shoes + taping vs. low-top shoes + untaped 6.50 vs. 33.40 *p* < 0.025
Sitler 1994 [71]	randomised controlled trial	semi-rigid ankle brace	1601 19.25 ± 1.36	primary and secondary prevention	ankle bracing significantly reduced the incidence of acute and contact-related ankle sprains, with no increase in knee injuries and no clear change in the severity of ankle injuries	Ankle sprain incidence density is reported as sprains per 1000 athlete-exposures (braced vs. unbraced): ankle sprain incidence density 1.60 vs. 5.20 *p* < 0.01 relative risk of ankle injury (previous vs. no previous sprain) 1.40 *p* > 0.30 knee injury risk: 9/812 vs. 8/789 *p* = 0.69
Moiler 2006 [124]	prospective non-randomised controlled trial	fibular repositioning taping (FRT) of the ankle	125 (m) 13–23	primary and secondary prevention	fibular repositioning taping significantly reduced the incidence of ankle sprain re-injury	Ankle sprain re-injury incidence rates are reported per 1000 exposures (FRT vs. no FRT): 8.90 vs. 43.00 OR 0.20, 95% CI [0.043–0.938], *p* = 0.041
McGuine 2011 [125]	randomised controlled trial	lace-up semi-rigid ankle brace	1460 (736 + 724) 16.00 ± 1.10	primary and secondary prevention	ankle bracing significantly reduced the incidence of acute ankle injuries, but not their severity, in both sexes for first-time injuries and re-injuries	Ankle injury incidence rates are reported per 1000 exposures (braced vs. unbraced): ankle injury incidence density 0.47 vs. 1.41 HR 0.32, 95% CI [0.20–0.52], *p* < 0.001 ankle re-injury incidence 0.83 vs. 1.79 HR 0.39, 95% CI [0.17–0.90], *p* = 0.028 effect independent of sex, previous ankle injury and BMI aHR 0.32, 95% CI [0.19–0.51], *p* < 0.001
Cusimano 2013 [149]	cross-sectional study	ankle bracing or taping	140 (46 + 94) 17.30 ± 3.30	perception and behavioural determinants of the use of ankle bracing or taping (protective external supports)	ankle bracing or taping use increased with coach enforcement, cost barrier, and perceived injury severity, while aesthetic appearance was the most common barrier, and previous ankle injury did not significantly predict use	Predictors of ankle brace or tape use: coach enforcement OR 35.71, 95% CI [10.01–127.36], *p* < 0.001 cost barrier OR 4.66, 95% CI [1.13–19.05], *p* < 0.05 perceived injury severity OR 2.77, 95% CI [1.04–7.37], *p* < 0.05 previous ankle injury OR 3.93, 95% CI [0.81–19.03], *p* > 0.05 Barriers to ankle brace or tape use: aesthetic appearance 29.30% overall; 27.70% among non-users (most common barrier)
Crockett 2015 [127]	prospective repeated-measures study	lace-up semi-rigid ankle brace	21 (13 + 8) 15.90 ± 0.83	biomechanics and functional performance	ankle bracing significantly improved dynamic postural control and single-leg functional performance, with no evidence of functional impairment	Star Excursion Balance Test at pre-, mid-, and post-season demonstrated improvement in all directions F = 34.00–40.62, all *p* < 0.001, pre- vs. post-season Cohen’s d 1.16–2.09 single-leg functional tests (triple crossover hop, vertical jump, and 6-m hop) showed significant time effects, with improvements from pre- to mid-, mid- to post-, and pre- to post-season F = 30.17–55.25, all *p* < 0.001
Klem 2017 [64]	controlled laboratory study	lace-up and hinged semi-rigid ankle brace	20 (f) 22.50 ± 3.80	biomechanics	ankle bracing significantly reduced ankle inversion during cutting without slowing movement or clearly restricting dorsiflexion or knee flexion	Bracing vs. unbracing: peak ankle inversion F (2, 38) = 4.50, *p* = 0.023, η^2^ = 0.190 MD = −1.70°, *p* = 0.023 movement velocity of the cut (hinged vs. lace-up vs. unbraced): 2.60 ± 0.40 m/s in all conditions, *p* = 0.877 no restriction in ankle dorsiflexion or knee flexion (braced vs. unbraced) all *p* > 0.05
Castro 2017 [128]	randomised controlled laboratory study	lace-up semi-rigid ankle brace	11 (m) 17.10 ± 0.10	biomechanics and performance	ankle bracing significantly reduced medial and lateral landing GRF without affecting vGRF or jump height	Bracing vs. unbracing: landing medial GRF peaks −15.70%, *p* = 0.035 landing lateral GRF peaks −24.90%, *p* = 0.012 vGRF and jump height all *p* > 0.05 timing of mediolateral GRF peaks all *p* > 0.05
Castro 2017 [145]	randomised controlled laboratory study	lace-up semi-rigid ankle brace	17 (m) 17.70 ± 1.40	biomechanics and performance	ankle bracing did not significantly impair ankle invertor or evertor torque, or the functional ratio, at any time point (pre-, mid-, or post-exercise)	Bracing vs. unbracing: ankle invertor and evertor peak torque, as well as the functional ratio, did not differ between conditions all *p* > 0.05 from pre- to post-exercise, ankle invertor peak torque showed changes of −17.70% (concentric) and −16.40% (eccentric), and ankle evertor peak torque of −15.10% and −15.20% in both conditions all *p* < 0.001, ES = 0.87–1.10 the functional ratio remained unchanged over time *p* = 0.80 heart rate remained similar 161.60 ± 8.20 vs. 161.20 ± 8.60 beats/min *p* = 0.816, ES = 0.05
Dewar 2019 [118]	randomised controlled laboratory study	semi-rigid ankle brace	16 (5 + 11) 26.94 ± 5.32	biomechanics	ankle bracing significantly reduced ankle and foot inversion during landing, while peak peroneus longus activity measured by surface electromyography (EMG) remained unchanged	Bracing vs. unbracing (dominant and non-dominant limbs): ankle braces reduced the degree of ankle inversion (means of maximum) 4.198 ± 2.970 and 4.945 ± 4.621 vs. 5.719 ± 3.984 and 6.568 ± 5.131 all *p* < 0.05 ankle braces reduced the degree of foot inversion in the rebounding task (means of maximum) 5.669 ± 2.885 and 4.086 ± 2.733 vs. 7.422 ± 3.229 and 7.048 ± 3.237 all *p* < 0.001 peak peroneus longus activity, measured by surface EMG during landing in millivolts, remained unchanged 0.1046 ± 0.0914 and 0.0790 ± 0.0241 vs. 0.0982 ± 0.0394 and 0.0795 ± 0.0270 all *p* > 0.05
Castro 2021 [129]	randomised clinical trial	lace-up semi-rigid ankle brace	11 (m) 17.10 ± 0.10	biomechanics and performance	ankle bracing significantly reduced mediolateral GRF without increasing vertical loading or decreasing jump height	Bracing vs. unbracing (percent MD): takeoff medial GRF peaks −16.00% across all periods all *p* < 0.001 landing lateral GRF peaks −9.00% across all periods all *p* < 0.001 landing medial and takeoff lateral GRF peaks −11.00% to −17.00% in periods 2–4 all *p* < 0.05 vGRF variables (peaks, impulses, loading rates) and jump height no main effect of condition all *p* > 0.05
Romero-Morales 2024 [68]	cross-sectional observational study	ankle taping	40 (20 + 20) 24.00 ± 6.00	biomechanics	ankle taping significantly reduced ankle dorsiflexion ROM in both males and females	Taped vs. untaped (percent MD): dorsiflexion ROM: −3.52% to −5.88% in men and −6.56% to −9.62% in women *p* = 0.001 η^2^ = 0.43–0.60

Note: %—percentage, ±—plus-minus, <—less than, >—greater than,−—minus, η^2^—eta-squared, °—degree, aHR—adjusted hazard ratio, beats/min—beats per minute, BMI—body mass index, CI—confidence interval, Cohen’s d—standardised effect size, EMG—electromyography, ES—effect size, f—female, F—F-statistic, FRT—fibular repositioning taping, GRF—ground reaction forces, HR—hazard ratio, m—male, m/s—metres per second, MD—mean difference, N—total sample size, OR—odds ratio, *p*—*p*-value, ROM—range of motion, vGRF—vertical ground reaction forces, vs.—versus.

## Data Availability

No new data were created or analysed in this study.

## Abbreviations

The following abbreviations are used in this manuscript:

η^2^	eta-squared
−	minus
%	percentage
+	plus
<	less than
>	greater than
±	plus-minus
°	degree
aHR	adjusted hazard ratio
beats/min	beats per minute
BMI	body mass index
CI	confidence interval
Cohen’s d	standardised effect size
DOI	Digital Object Identifier
DT	dynamic tape
EAB	elastic adhesive bandage
Embase	Excerpta Medica Database
EMG	electromyography
ES	effect size
et al.	and others (lat. et alia)
f	female
F	F-statistic
FRT	fibular repositioning taping
GRF	ground reaction forces
HR	hazard ratio
KT	kinesio tape/taping
lat.	Latin
m	male
m/s	metre per second
MD	mean difference
MEDLINE	Medical Literature Analysis and Retrieval System Online
mid	mid-season
N	total sample size
NY	New York
OR	odds ratio
OSF	Open Science Framework
*p*	*p*-value (probability value)
post	post-season
pre	pre-season
ROM	range of motion
SFA	Sport for All
UK	United Kingdom of Great Britain and Northern Ireland
USA	United States of America
vGRF	vertical ground reaction forces
vs.	versus
WHO	World Health Organisation
WoS CC	Web of Science Core Collection
